# Investigating the mediating roles of psychological resilience and digital addiction in the relationship between emotional intelligence and mental health: a meta-analytic structural equation modeling approach

**DOI:** 10.3389/fpsyg.2026.1780246

**Published:** 2026-05-26

**Authors:** Turgut Karaköse, Tijen Tülübaş, Bünyamin Han, Sedat Kanadli, Mehmet Ozdogru, Abdurrahman Kardas, Nurcihan Aslan

**Affiliations:** 1 Faculty of Education, Kütahya Dumlupinar University, Kütahya, Türkiye; 2 Faculty of Education, Mersin University, Mersin, Türkiye; 3 Provincial Directorate of National Education, Batman, Türkiye; 4 Hamidiye Vocational School of Health Services, University of Health Sciences, Istanbul, Türkiye

**Keywords:** behavioral addiction, digital addiction, emotional intelligence, mental health, psychological resilience, university students

## Abstract

**Introduction:**

Research shows that university students are increasingly becoming more prone to digital addiction, leading to serious mental health problems. Emotional intelligence and psychological resilience, on the other hand, are considered to play a role in their likelihood of experiencing digital addiction and mental health problems. Studies investigating the interrelationships between these constructs are rare despite their growing significance in the contemporary digital world. Therefore, this study aims to investigate the dual and multiple relationships between these variables by testing a hypothetical research model.

**Method:**

This study uses meta-analytic structural equation modeling (MASEM) analysis to test the structural relationships in the study model. The study also tests some alternative models regarding the relationships among the study variables to offer a more nuanced understanding. Data for the study were collected step-wise from Web of Science (WoS), Scopus, and Google Scholar. Data analysis was conducted using correlation coefficients gathered from 257 studies included in the dataset.

**Results:**

By quantitatively synthesizing results from 257 studies, the study showed that university students' emotional intelligence can be associated with mental health problems both directly and indirectly through digital addiction and psychological resilience.

**Discussion:**

By testing an integrated model of emotional, psychological, and behavioral factors within a single exploratory framework, the study provided a more comprehensive account of mental health functioning among university students and suggested important implications for promoting university students' mental health.

## 1 Introduction

Advancements in digital technologies continue to reshape many aspects of daily life (Liţan, 2025; Qian et al., 2024) from patterns of communication to socialization, entertainment, or learning. Despite many conveniences they provide, they bear significant risks to human health (Kuss and Griffiths, 2017) through interacting with several psychological processes such as identity development, attachment styles, or emotion regulation mechanisms (Monacis et al., 2017), as well as their addictive potential.

In recent years, excessive exposure to or problematic use of digital technologies, namely digital addiction, has been classified as a behavioral addiction because it is likely to cause impairments in daily life, difficulties in impulse control, and a significant decline in social relationships (Griffiths, 2005; Kardefelt-Winther, 2014). Studies have shown that digital addiction is strongly associated with various psychological risks such as depression, anxiety, stress, loneliness, low self-esteem, attention problems, and sleep disorders (Lazarotto Schroeder et al., 2023; Kircaburun and Griffiths, 2019; Sohn et al., 2019).

The mental health literature indicates that digital addiction is not only linked to psychological wellbeing but also affects executive functions, attention processes, and social relationships in a multifaceted way. For example, studies on social media addiction, a subtype of digital addiction, reveal that students' constant desire to be online increases their tendency for social comparison, which can later trigger depressive symptoms (Appel et al., 2016; Yan et al., 2024). Similarly, intensive smartphone use is associated with difficulties in emotion regulation and increased stress in individuals (Hormes et al., 2014; Vahedi et al., 2021). Furthermore, sleep disruption is associated with digital addiction, suggesting that late-night screen exposure suppresses melatonin secretion and leads to a decrease in both sleep duration and quality (Exelmans and Van den Bulck, 2016; Li et al., 2020). Existing evidence suggests that digital addiction can influence mental health through many interconnected underlying mechanisms (Shiferaw et al., 2025).

Research indicates that university students are more prone to digital addiction compared to other age groups (Amano et al., 2023; Boursier et al., 2020; Can and Onan, 2023; Crowhurst and Hosseinzadeh, 2024; Kuss and Griffiths, 2017; Ruckwongpatr et al., 2022; Sanchez-Fernandez and Borda-Mas, 2023; Servidio, 2021). Empirical results suggest that university students' search for social belonging (Sanchez-Fernandez and Borda-Mas, 2023), academic stress and anxiety (Crowhurst and Hosseinzadeh, 2024), search for social approval and pressure to present oneself (Casale and Banchi, 2020), having more free time and constant access to personal devices (Sarfo et al., 2023) as well as the influence of digital platforms on their identity formation processes (Soh et al., 2024) are some of the significant reasons for the increased risk of digital addiction among university students. Furthermore, research findings suggest that online education practices and the increasing need for digital communication can also trigger digital addiction among university students (Besalti and Satici, 2022; Sarfo et al., 2023).

Studies focusing on the link between university students' digital addiction and mental health reveal a significant negative correlation between psychological resilience and digital addiction (Bertocchi et al., 2022; Elhai et al., 2017), indicating that students with high psychological resilience are better at coping with real-life stressors and therefore less likely to resort to escapist coping strategies through excessive digital media consumption (Zhao et al., 2025). Similarly, university students with higher emotional intelligence are observed to have a lower risk of digital addiction and mental health problems (Reyes-Wapano, 2021; Zeidner et al., 2012) due to having higher psychological resilience and a greater capacity to cope with negative emotions (Färber and Rosendahl, 2018). To conclude, emotional intelligence and psychological resilience are considered to play a role in reducing mental health problems associated with digital addiction (Nadeem et al., 2025; Shuo et al., 2022).

Taking digital addiction as an umbrella term encompassing problematic use of digital technologies such as the internet, smartphone, social media, AI-apps, online gaming, or digital communication devices (Basel et al., 2020; Sohn et al., 2019), this study aims to examine the interrelationships between emotional intelligence, digital addiction, psychological resilience, and mental health problems in university students within a more holistic framework. Using data from previous research on dual or multiple relationships among the variables, the study analyzes how university students' emotional intelligence affects their mental health both directly and indirectly through digital addiction and psychological resilience. To this end, we employed a meta-analytic structural equation modeling approach (MASEM), which combines meta-analysis and structural equation modeling to test a theoretical model using aggregated evidence from many studies. MASEM goes beyond traditional meta-analyses, which are limited to isolated effect sizes and do not capture interdependencies among several variables, by simultaneously estimating direct and indirect (mediated) relationships among study variables and examining relative effects and indirect pathways within a unified theoretical system, and thus testing a theory-driven system of relationships rather than separate bivariate links. Therefore, by integrating emotional intelligence, mental health, digital addiction, and psychological resilience within a single MASEM framework, the present study moves beyond fragmented evidence to provide a coherent test of their joint functioning, enabling the examination of interdependencies and indirect pathways linking emotional intelligence to mental health through digital addiction and psychological resilience within a unified theoretical model.

## 2 Theoretical framework and the hypotheses

### 2.1 Emotional intelligence and mental health problems

Emotional intelligence refers to an individual's emotional strength and capability to regulate their own and others' emotions (Ciarrochi et al., 2002). Research has shown that people with higher emotional intelligence are better able to protect themselves from the negative effects of stress, reporting less depression, hopelessness, and suicidal ideation (Akerjordet and Severinsson, 2004; Faghirpour et al., 2011; Reyes-Wapano, 2021; Zeidner et al., 2012), suggesting that emotional intelligence can act as a mechanism to prevent mental health problems (Shabani et al., 2010; Van Dusseldorp et al., 2011). It is often argued that negative emotional states are associated with unhealthy physiological functioning patterns (Shabani et al., 2010). Similarly, all three dimensions of emotional intelligence, wellbeing, self-control, and sociability, have been shown to positively influence mental health (Fernández-Abascal and Martín-Díaz, 2015). Based on these findings, we propose our first hypothesis:

*H1. University students' emotional intelligence has a significant negative effect on their mental health problems*.

### 2.2 Emotional intelligence and digital addiction

Research indicates a relationship between individuals' emotional intelligence levels and digital addiction behaviors (Alaswad et al., 2025; Biolcati et al., 2025). Individuals with low emotional intelligence may be more prone to internet addiction, while those with high emotional intelligence are less inclined to excessive internet use and its negative consequences (Yu and Zhou, 2021). Studies have shown a significant negative correlation between emotional intelligence and internet addiction (Ahmed et al., 2025; Zewude et al., 2024; Biolcati et al., 2025), suggesting that higher levels of emotional intelligence can reduce internet addiction among university students (Saraiva et al., 2018; Fernández-Martínez et al., 2023). Based on this evidence, we propose our second hypothesis:

*H2*. *University students' emotional intelligence has a significant negative effect on their digital addiction*.

### 2.3 Emotional intelligence and psychological resilience

Emotional intelligence and psychological resilience are interrelated psychological concepts associated with several behavioral outcomes (Abualruz et al., 2024; Kökçam et al., 2022; Olaleye and Lekunze, 2024). Emotional intelligence enhances empathy, responsiveness to others' emotions, and emotional self-regulation by supporting an individual's stress responses and coping strategies (Li et al., 2015), which can, in turn, support psychological resilience. It has also been reported that emotional intelligence affects students' wellbeing both directly and through enhancing psychological resilience (Akbari and Khormaiee, 2015; Bano and Pervaiz, 2020), underscoring the critical role of developing emotional intelligence and psychological resilience in educational settings (Panagiotidou et al., 2025). Similarly, Kartol et al. (2024) showed a positive correlation between emotional intelligence and psychological resilience in university students. Based on this evidence, we propose our third hypothesis:

*H3. University students' emotional intelligence has a significant positive effect on their psychological resilience*.

### 2.4 Digital addiction and mental health problems

While digital technologies offer various benefits for young people, their problematic and extensive use bears significant risks (Elhai et al., 2017; Matar Boumosleh and Jaalouk, 2017; Bertocchi et al., 2022). Students' excessive use of digital technologies such as smartphones or social media can result in addiction (Aboujaoude and Gega, 2020; Singh and Singh, 2019; Cerniglia et al., 2017). Meta-analytic studies report a high correlation between problematic digital technology use and various mental health problems such as depression, anxiety, and sleep disorders (Bertocchi et al., 2022; Feng et al., 2022; Chang et al., 2022; Elhai et al., 2017; Meng et al., 2022; Dresp-Langley and Hutt, 2022; Karakose et al., 2023).

In their study with university students, Machado de Oliveira et al. (2023) found that increased smartphone use and high addiction rates worsened their quality of life and caused several mental health problems. Several other studies have revealed that problematic and pathological internet use is associated with various psychopathological symptoms (Carli et al., 2012; Çelik and Odaci, 2013). In their research, Shiferaw et al. (2025) found that young people with digital addiction reported lower self-ratings of mental health. Similarly, Zhao et al. (2025) reported that digital addiction had a negative impact on the physical and mental health of university students. Based on these findings, we propose our fourth hypothesis:

*H4. University students' digital addiction has a significant positive effect on their mental health problems*.

### 2.5 Psychological resilience and mental health problems

The term resilience is used to describe various phenomena ranging from preventing mental health problems to successful adaptation and rapid recovery after experiencing life challenges such as post-traumatic psychological growth (Rutten et al., 2013). Similarly, psychological resilience refers to the process of multiple biological, psychological, social, and ecological systems interacting to help individuals regain, maintain, or improve their mental wellbeing when faced with one or more risk factors (Ungar and Theron, 2020). Empirical evidence suggests that psychological resilience can act as a regulatory mechanism for mental health (Güldaş and Karsli, 2023). For instance, Färber and Rosendahl (2018) found that lower levels of psychological resilience were associated with somatic illnesses, while Afek et al. (2021) revealed a significant correlation between psychological resilience and mental health. Based on these results, we propose our fifth hypothesis:

*H5. University students' psychological resilience has a significant negative effect on their mental health problems*.

### 2.6 Psychological resilience and digital addiction

A significant negative correlation has been found between digital addiction and psychological resilience (Cao et al., 2022; Kocabiyik and Bacioglu, 2022; Korkut and Akman, 2024), and higher psychological resilience is said to reduce behaviors that lead to digital addiction (Wang, 2025). It is predicted that individuals with high psychological resilience reduce their tendency toward excessive digital media consumption (Zhao et al., 2025), while low psychological resilience increases susceptibility to digital escapism and behavioral addiction (Reji, 2025). Özsavran and Ayyildiz (2024) found that digital game addiction led to lower levels of psychological resilience, suggesting that psychological resilience can predict problematic internet use or internet addiction (Ataman-Bor et al., 2025). Based on this evidence, we propose our sixth hypothesis:

*H6. University students' digital addiction has a significant negative relationship with their psychological resilience*.

### 2.7 Mediating roles of digital addiction and psychological resilience

Emotional intelligence is negatively correlated with behavioral addictions and plays both a moderating and mediating role in the relationships between behavioral addictions and mental health problems (Biolcati et al., 2025). Emotional intelligence can affect students' mental health both directly (Reyes-Wapano, 2021; Zewude et al., 2024) and indirectly by moderating their tendency to develop digital addiction. Thus, we propose our seventh hypothesis as follows:

*H7. Digital addiction mediates the relationship between emotional intelligence and mental health problems*.

Psychological resilience is considered a sign of a healthy psychological state and may partially mediate the relationship between emotional intelligence and mental health problems. Emotional intelligence can support psychological resilience by enhancing individuals' ability to manage stress and cope with problems (Akbari and Khormaiee, 2015; Jayalakshmi and Magdalin, 2015). Considering the positive effects of psychological resilience on mental health (Al Eid et al., 2020; Güldaş and Karsli, 2023; van der Meulen et al., 2018), psychological resilience can also mediate the relationship between emotional intelligence and mental health problems. Thus, we propose our eighth hypothesis as follows:

*H8. Psychological resilience mediates the relationship between emotional intelligence and mental health problems*.

**Figure 1:**
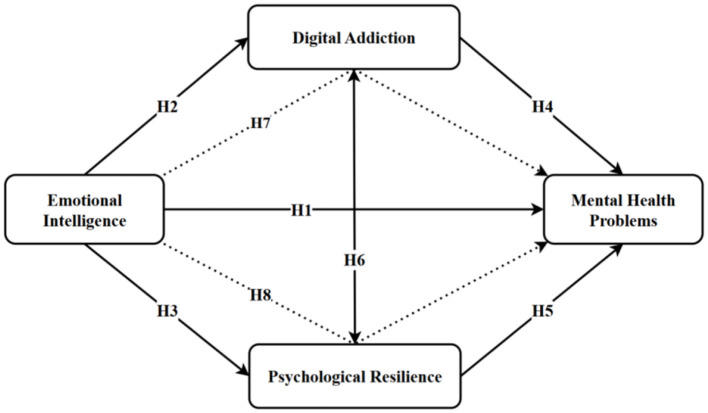
The proposed research model.

Within the context of existing evidence regarding the relationships among variables and the hypotheses developed from this evidence, we developed the research model presented in Figure 1.

## 3 Methodology

This study employs meta-analytic structural equating modeling (MASEM) to test the relationships in the hypothesized model on emotional intelligence, digital addiction, psychological resilience, and mental health problems. The analyses are conducted using the Pearson correlations gathered from 257 studies on the particular variables involved in the model (Jeyaraj and Dwivedi, 2020).

### 3.1 Data collection

**Figure 2:**
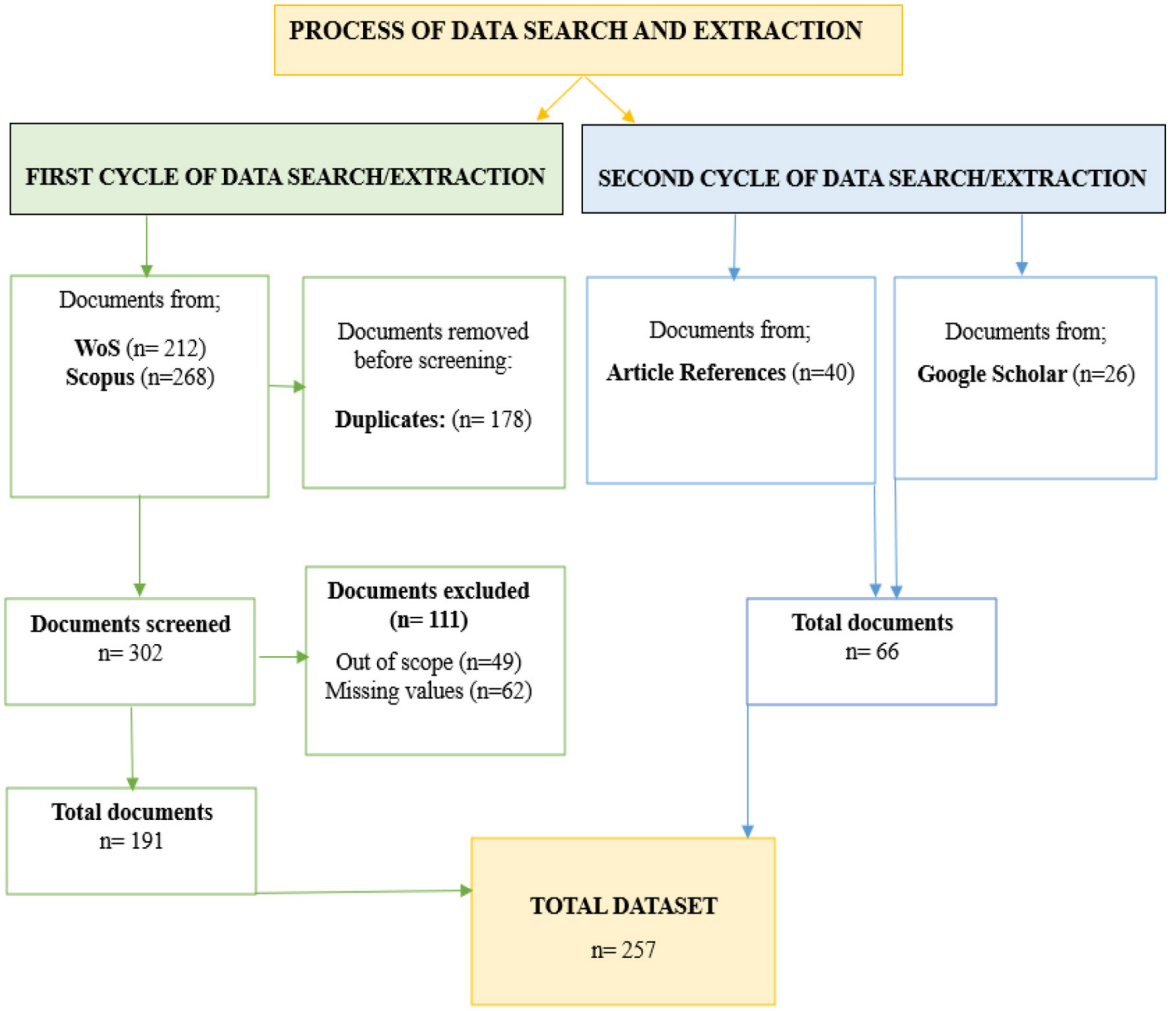
The PRISMA diagram.

Data for the study were collected step-wise from Web of Science (WoS), Scopus, and Google Scholar. The initial search was conducted on 28 September, 2025 on WoS and Scopus using a combination of study variables, as well as the other types of digital addictions, such as smartphone, social media, or video-game addiction. This search yielded 212 documents from WoS and 268 from Scopus. Following the psychological PRISMA 2020 guidelines (Page et al., 2021), we employed a detailed data extraction (Figure 2).

Two independent reviewers from among the researchers conducted the whole data search and extraction process, which was later double-checked by two other independent reviewers from among the researchers. The following inclusion/exclusion criteria was applied during data extraction:

INCLUDE: peer-reviewed articles reporting Pearson correlations in the context of university students; articles written in English.

EXCLUDE: thesis, books (book chapters or conference proceedings; qualitative, review, conceptual studies; quantitative studies not reporting Pearson correlation; K-12 or general population context; non-English articles.

First, we compared the WoS and Scopus documents and identified 178 duplicate records. Next, we scanned through the titles and abstracts of the remaining 302 documents to identify documents falling out of our research scope (e.g., focusing on K-19 students, adults, etc.), and 49 documents were removed for this reason. Next, we scanned through the results sections of the remaining 253 documents and extracted 62 studies to obtain the Pearson correlation values. At this stage, we also skimmed the references of these articles and identified 40 additional papers that were missed earlier. Finally, we conducted a final search on Google Scholar to identify any recent research that might not have been indexed on the two databases yet. We identified 26 documents to be included in the dataset. Consequently, a total of 257 documents were included in the final dataset.

### 3.2 Data analysis

A two-stage structural equation modeling approach was used to determine the overall effect size of the relationship between variables and to test the goodness-of-fit of the constructed models (Cheung, 2014, 2015), using Pearson's correlation coefficients extracted from the 257 studies. In the first stage, a total correlation matrix is created from the correlation matrices extracted from the primary studies. For this purpose, correlation matrices were combined according to the random effects model to calculate the aggregate correlation matrix and the variance-covariance components (Tau^2^) representing inter-study heterogeneity. This calculation was performed using the full information maximum likelihood (FIML) estimation method. In the second stage, structural equation models are constructed using this matrix to assess model-data fit. At this stage, parameter estimation was performed using the weighted least squares (WLS) method, where the inverse of the asymptotic sampling covariance matrix obtained from the first stage was used as the weighting matrix. webMASEM, a web-based software, was used for this analysis (Jak et al., 2021). First, correlation matrices were combined according to the random effects model (Borenstein et al., 2009) to determine the overall effect size and the magnitude of heterogeneity (*I*^2^) of the relationship between the variables. The overall effect sizes were calculated using Funder and Ozer's (2019) scale according to which heterogeneity up to 0.10 is “very small”, up to 0.20 “small”, up to 0.30 “medium”, up to 0.40 “wide”, and above 0.40 “very wide”. The magnitude of heterogeneity up to 25% was evaluated as “small”, up to 50% “medium”, and up to 75% “high” (Higgins et al., 2003). To determine whether publication bias had any effect on the calculated mean effect sizes, we used the funnel plot, and performed Egger's regression test (Egger et al., 1997) to determine if the asymmetry in the funnel plot was significant. When the result of this test shows no significant asymmetry, this indicates no statistically significant effect of publication bias. Next, model-data fits were evaluated for both the proposed and alternative models by calculating the RMSEA, SRMR, CFI, and TLI indices. An RMSEA value equal to or below 0.05 was interpreted as “excellent” fit; a value between 0.05 and 0.08 was interpreted as acceptable fit (Schermelleh-Engel et al., 2003). A SRMR value of 0.08 or less is considered an excellent “fit,” while a value between 0.08 and 0.1 is considered an acceptable fit (Kline, 2023). For comparative fit indices, TLI and CFI values >0.95 indicate an “excellent fit,” while values between 0.90 and 0.95 indicate an acceptable fit (Kline, 2023). Furthermore, AIC and BIC values were examined to determine which model best fit the data, and the model with the lowest AIC and BIC values was identified as the best-fit model (Schermelleh-Engel et al., 2003).

## 4 Results

### 4.1 Summary effects

Correlation matrices were constructed by combining the correlation coefficients extracted from studies in the dataset according to the random effects model. The calculated effect sizes are presented in Table 1.

**Table 1:** Summary of MASEM analysis.

Associations	*k*	Total sample	*r*	%95 Confidence Interval		*I^2^*	Egger's Sig.
				Lower Limit	Upper Limit		
DA&MH	103	65967	0.338	0.308	0.368	%93.9	0.275
DA&PR	51	30463	−0.264	−0.295	−0.233	%86.6	0.388
DA&EI	42	21242	−0.316	−0.371	−0.260	%95.1	0.237
MH&PR	78	45384	−0.383	−0.414	−0.351	%93.1	0.257
MH&EI	44	21254	−0.341	−0.397	−0.285	%95.9	0.925
PR&EI	46	17737	0.512	0.459	0.565	%96.9	0.897

DA, digital addiction; MH, mental health; PR, psychological resilience; EI, emotional intelligence.

### 4.2 Test of the mediation analysis

The effect size between digital addiction and mental health problems was calculated as *r* = 0.338, 95% CI[0.308, 0.368], which is interpreted as “large”. The effect size between digital addiction and emotional intelligence was calculated as *r* = −0.316, 95% CI[−0.371, −0.260], which is negative and “large”. The effect size between digital addiction and psychological resilience was calculated as *r* = −0.264, 95% CI[−0.295, −0.233], which is negative and “moderate”. The effect size between emotional intelligence and mental health problems was calculated as *r* = −0.341, 95% CI [−0.397, −0.285], while the correlation effect size between psychological resilience and mental health problems was calculated as *r* = −0.383, 95% CI[−0.414, −0.351], showing mental health problems are negatively and largely correlated with emotional intelligence and psychological resilience. A significant “moderate” correlation (*r* = 0.270, 95% CI[0.143, 0.398]) was found between emotional intelligence and psychological resilience. *I*^2^ values range from 86.6% to 96.9%, indicating a “high” level of variance between effect sizes, suggesting a significant portion of the variation in effect sizes may stem from the specific characteristics of the studies included in the meta-analysis. A regression analysis was conducted to examine the effect of publication bias on mean effect sizes, which revealed that asymmetry in the funnel plots (Appendix) was not statistically significant at the 95% confidence interval (*p* < 0.05). This result indicates that publication bias is not statistically significant, and therefore the calculated mean effect sizes are valid and reliable.

#### 4.2.1 Test of the proposed model

The correlation matrices obtained from studies were combined to analyze the model-data fit of the proposed research model. The calculated goodness-of-fit indices are given in Table 2.

**Table 2:** The goodness-of-fit indices for the proposed model.

Model	χ2(df)	*p*-value	RMSEA	RMSEA %95 Li	RMSEA %95 Ui	SRMR	TLI	CFI	AIC	BIC
Original Model	9.69(1)	0.002	0.008	0.004	0.013	0.035	0.973	0.996	7.689	−2.138

**Figure 3:**
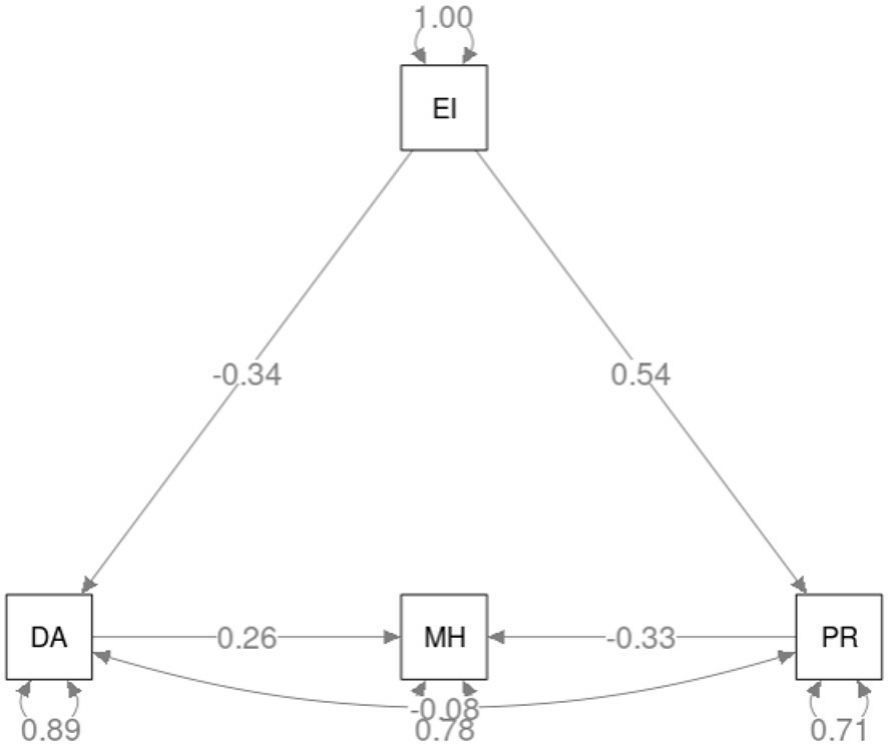
The path graph of the proposed research model. (DA, digital addiction; MH, mental health; PR, psychological resilience; EI, emotional intelligence).

The chi-square test for the Proposed Model was not statistically significant (χ2 = 9.689, *p* > 0.05). RMSEA showed an “excellent” fit with a 95% CI of 0.008 [0.004, 0.013]; SRMR showed a “good” fit with a value of 0.035; TLI showed an “excellent” fit with a value of 0.973, and CFI showed an “excellent” fit with a value of 0.996. Taken together, the model shows a good model-data fit. The path graph of the proposed research model is shown in Figure 3.

As shown in Figure 3, emotional intelligence had a large negative effect (β = 0.335, 95% CI = [−0.389, −0.281]) on digital addiction. Similarly, emotional intelligence had a large positive effect on psychological resilience (β = 0.535, 95% CI [0.484, 0.586]). Emotional intelligence explains 11% of the variance in digital addiction and 29% of the variance in psychological resilience, indicating that emotional intelligence can significantly influence mental health and psychological resilience. Digital addiction had a moderate positive effect on mental health problems (β = 0.258, 95% CI [0.223, 0.292]) while psychological resilience had a large negative effect (β = −0.328, 95% CI [−0.362, −0.293]). These findings indicate that digital addiction can increase mental health problems, while psychological resilience can reduce them. Digital addiction and psychological resilience explain 22% of the variance in mental health problems.

**Figure 4:**
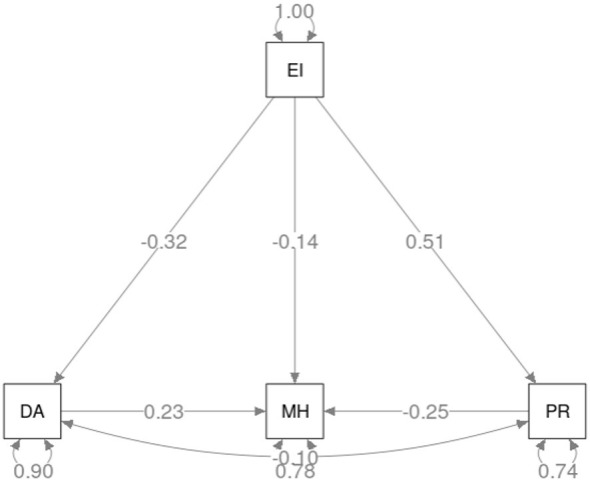
The path graph of the mediation model. (DA, digital addiction; MH, mental health; PR, psychological resilience; EI, emotional intelligence).

Digital addiction and psychological resilience have a very small negative correlation (*r* =-0.080, 95% CI = [−0.125, −0.036]), and both mediate the relationship between emotional intelligence and mental health problems. To determine whether these variables are full or partial mediators, a regression arrow was drawn from emotional intelligence to mental health problems. The path graph of the mediation model is shown in Figure 4.

Because the mediating model became saturated (df = 0), no goodness-of-fit index was calculated. The regression coefficient between emotional intelligence and mental health problems was negative, small, and significant (β = −0.141, 95% CI = [−0.227, −0.056]). Accordingly, digital addiction and psychological resilience are partial mediators in the relationship between emotional intelligence and mental health problems. In other words, the effect of emotional intelligence on mental health may occur both directly and indirectly through digital addiction and psychological resilience, indicating that emotional intelligence can contribute to the resolution of mental health problems by reducing digital addiction and strengthening psychological resilience.

### 4.3 Test of alternative models

Three alternative models to the proposed research model were created and tested to identify whether any better model fit could be obtained. The calculated goodness-of-fit indices are given in Table 3.

**Table 3:** Goodness-of-fit indices for the alternative models.

Model	χ2(df)	*p*-value	RMSEA	RMSEA %95	RMSEA %95	SRMR	TLI	CFI	AIC	BIC
Model 1	99.90(1)	0.000	0.027	0.023	0.031	0.094	0.690	0.949	97.9	88.07
Model 2	199.97(2)	0.000	0.027	0.024	0.030	0.095	0.691	0.897	196	176.3
Model 3	131.65(2)	0.000	0.022	0.019	0.025	0.120	0.798	0.933	127.7	108
Model 4	316.61(2)	0.000	0.034	0.031	0.037	0.132	0.509	0.836	312.6	293

**Figure 5:**
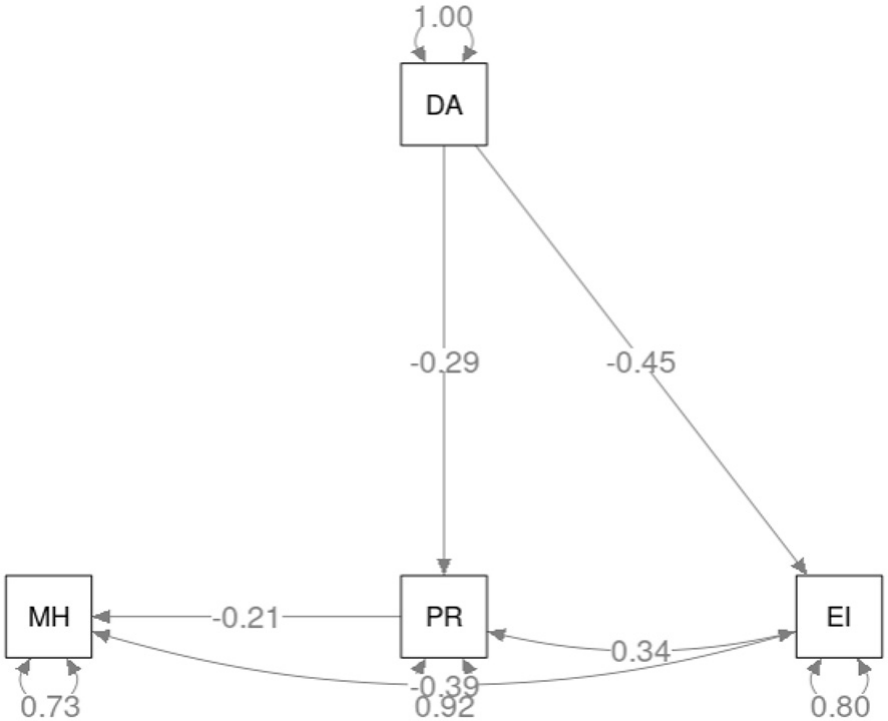
The path graph of the alternative model 1. (DA, digital addiction; MH, mental health; PR, psychological resilience; EI, emotional intelligence).

In the first alternative model (Model 1), the independent variable of the proposed model (emotional intelligence) was exchanged with the dependent variable of digital addiction. The path graph of the alternative model 1 is shown in Figure 5.

All regression coefficients of Model 1 were found to be significant at the 95% confidence level (*p* < 0.05). Considering the goodness-of-fit indices of Model 1 given in Table 3, the RMSEA value of 0.027 95% [0.023, 0.032] indicates an “excellent” fit. In contrast, the CFI value of 0.949 indicates an “acceptable” fit. The SRMR value of 0.949 is within the acceptable range. In contrast, the TLI value of 0.691 is well below the acceptable level. TLI penalizes complex, or less restrictive, models with a downward correction, while parsimonious, or more restrictive models are rewarded with an increase in the fit index (Schermelleh-Engel et al., 2003). Therefore, the TLI being below the acceptable level may have resulted from the model having a low degree of freedom (df = 1), implying that this model fits the data and validly explains the structural relationships between the variables.

**Figure 6:**
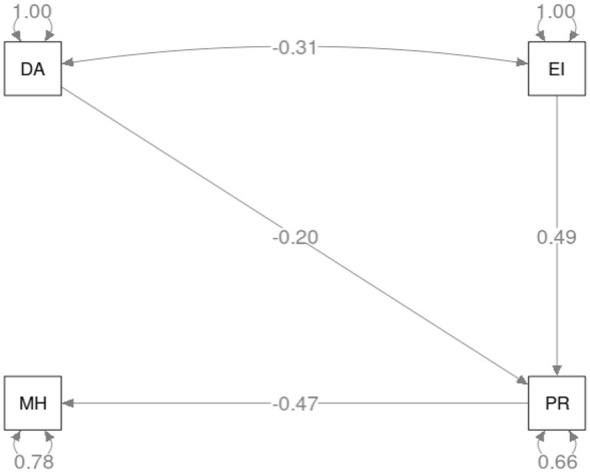
The path graph of the alternative model 2. (DA, digital addiction; MH, mental health; PR, psychological resilience; EI, emotional intelligence).

To increase the degrees of freedom and improve the TLI value, models with multiple independent variables (df = 2) were established. In Model 2, digital addiction and emotional intelligence were considered as independent variables, and both were determined as predictors of psychological resilience, which in turn predicts mental health problems. The path graph of alternative model 2 is shown in Figure 6.

All regression coefficients of Model 2 were found to be significant at the 95% confidence level (*p* < 0.05). A comparison between the goodness-of-fit indices of Model 1 and Model 2 reveals that the RMSEA (0.027), SRMR (0.095), and TLI (0.691) indices of Model 2 have similar values to the indices of Model 1. However, the CFI index for Model 2 remains below the “acceptable” limits, indicating that Model 2 does not fit the data.

**Figure 7:**
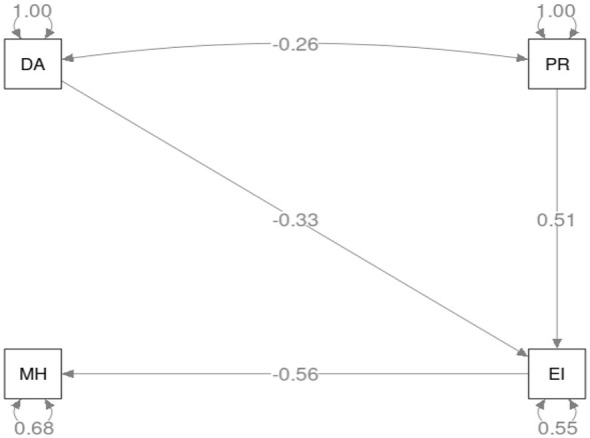
The path graph of the alternative model 3. (DA, digital addiction; MH, mental health; PR, psychological resilience; EI, emotional intelligence).

Unlike Model 2, the positions of emotional intelligence and psychological resilience were reversed in Model 3, with emotional intelligence serving as the mediating variable. The path graph of the alternative model 3 is shown in Figure 7.

All regression coefficients of Model 3 were statistically significant at the 95% confidence level (*p* < 0.05). The RMSEA (0.019) for Model 3 showed an excellent fit, but SRMR (0.12) was below the acceptable limit. Although the TLI (0.798) value increased, it remained below the acceptable limit. Similarly, CFI (0.933) increased slightly, reaching an “acceptable” fit level. These results imply that the model-data fit is not achieved for Model 3, so it is insufficient to explain the structural relationships between the variables.

**Figure 8:**
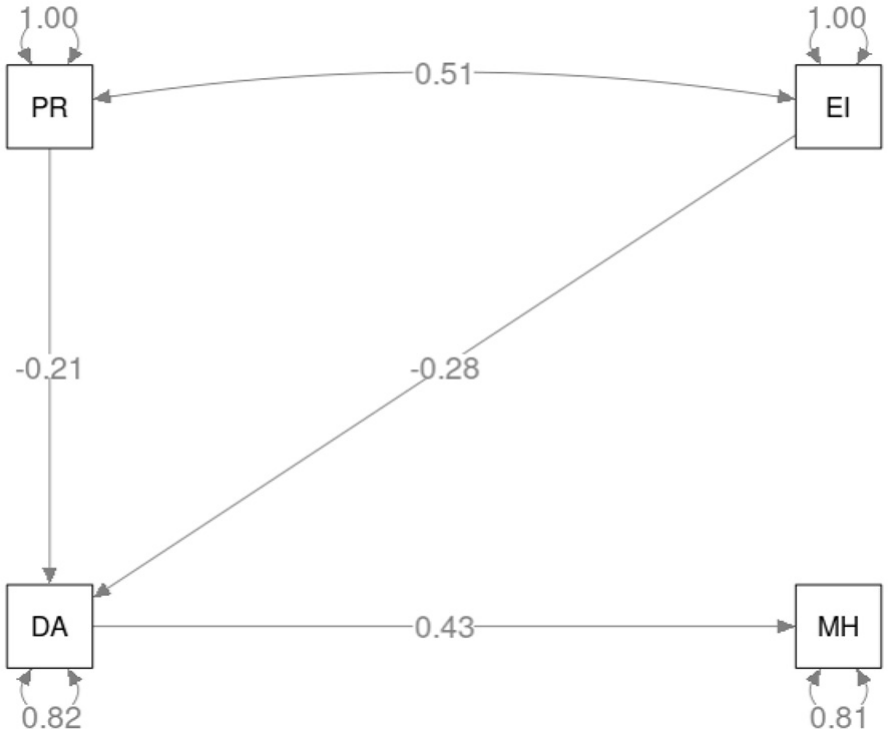
The path graph of the alternative model 4. (DA, digital addiction; MH, mental health; PR, psychological resilience; EI, emotional intelligence).

Finally, Model 4 was constructed, in which the positions of digital addiction and emotional intelligence were swapped, with digital addiction as the mediating variable. The path graph of alternative model 4 is shown in Figure 8.

All regression coefficients in Model 3 were statistically significant at the 95% confidence level (*p* < 0.05). However, all indices except RMSEA (0.034) (SRMR, TLI, and CFI) were well below acceptable levels, indicating that Model 4 did not fit the data well.

One way to determine the best model to explain the structural relationships between the variables is to check the AIC and BIC indices. A model with lower AIC and BIC indices is considered to fit the data best (Kline, 2023). Accordingly, the AIC (=7.689) and BIC (=-2.14) calculated for the Proposed Model were the lowest among the models. Based on these results and the goodness-of-fit indices calculated for the proposed model, it can be said that the proposed model best explains the relational structure in the dataset. Among the alternative models, Model 1 (AIC = 97.9; BIC = 88.1) is the most suitable model for the data. The fundamental difference between the Proposed Model and Model 1 lies in whether the independent variable is emotional intelligence or digital addiction. Accordingly, the fact that both models fit the data indicates that emotional intelligence can reduce digital addiction and vice versa.

## 5 Discussion

This study employed a novel and advanced methodology, (MASEM), to examine the dual and multifaceted relationships among emotional intelligence, psychological resilience, digital addiction, and mental health problems among university students in a single theoretical model. Using the proposed research model and other alternative models developed during structural equation analysis, the analyses yielded comprehensive and generalizable results that will contribute to the relevant literature and guide practitioners.

Analyses have shown that higher levels of emotional intelligence can be linked to reduced levels of digital addiction and mental health problems in university students. Research showed that individuals with low emotional intelligence are more prone to internet addiction, while those with high emotional intelligence are less inclined to excessive internet use and its associated negative consequences (Alaswad et al., 2025; Ahmed et al., 2025; Biolcati et al., 2025; Yu and Zhou, 2021; Zewude et al., 2024). Specifically, high emotional intelligence has been associated with lower levels of internet addiction among university students (Fernández-Martínez et al., 2023; Saraiva et al., 2018). Similarly, individuals with improved ability to regulate their own emotions and the emotions of others are more protected against the negative effects of stress, thus having a lower probability of developing depression, hopelessness, and suicidal ideation (Ciarrochi et al., 2002). In other words, emotional intelligence, with all three dimensions of wellbeing, self-control, and sociability, has been closely linked to improved mental health (Akerjordet and Severinsson, 2004; Faghirpour et al., 2011; Fernández-Abascal and Martín-Díaz, 2015; Reyes-Wapano, 2021; Zeidner et al., 2012). Regarding psychological resilience, existing evidence shows that emotional intelligence strengthens coping and self-regulation skills by supporting adaptive responses in stressful situations, thus significantly contributing to psychological resilience (Armstrong et al., 2011; Li et al., 2015). Studies in the educational context have found positive and significant relationships between emotional intelligence and psychological resilience among university students, emphasizing the importance of developing these two constructs together (Kartol et al., 2024; Panagiotidou et al., 2025).

Our study adds to the existing evidence by showing that digital addiction among university students can be linked to mental health problems (Bertocchi et al., 2022; Elhai et al., 2017; Matar Boumosleh and Jaalouk, 2017). Meta-analytic studies reveal strong associations between problematic digital technology use and psychological problems that can lead to mental health problems such as depression, anxiety, and sleep disorders (Bertocchi et al., 2022; Dresp-Langley and Hutt, 2022; Elhai et al., 2017; Karakose et al., 2023). Studies, particularly those conducted on university students, highlight the negative influence of digital addiction on quality of life and mental health (Çelik and Odaci, 2013; Machado de Oliveira et al., 2023). Key reasons why digital addiction is a significant risk factor for young people's mental health include using technology to escape negative emotions, exposure to intense social comparison on social media, and damage to self-image (Sun and Zhang, 2021; Yue et al., 2022). Furthermore, excessive screen use disrupts sleep patterns (Tahir et al., 2021; Bertocchi et al., 2022), makes time management difficult (Han et al., 2023), reduces face-to-face social interaction (Li et al., 2021), and increases feelings of loneliness. Declines in academic functioning (Machado de Oliveira et al., 2023), decreased physical activity (Aziz et al., 2021; Zhao et al., 2025), and developmental vulnerabilities of adolescents and young adults (Xu et al., 2021) can also lead to both digital addiction and mental health problems, such as anxiety and depression (Lazarotto Schroeder et al., 2023; Sohn et al., 2019).

The study revealed a correlation between psychological resilience and digital addiction. Previous studies have shown that individuals with high levels of psychological resilience are better able to control behaviors leading to digital addiction (Wang, 2025), develop alternative coping strategies instead of using digital environments as a means of emotional relief (Färber and Rosendahl, 2018; Aslan et al., 2025), and thus are less likely to enter the addiction cycle (Rutten et al., 2013; Xie et al., 2023). Conversely, low psychological resilience can increase individuals‘ tendency to use digital environments to cope with stress, loneliness, and negative emotions, raising the risk of digital escapism (Sun and Zhang, 2021) and behavioral addiction (Yue et al., 2022; Reji, 2025). Indeed, recent research (Özsavran and Ayyildiz, 2024; Ataman-Bor et al., 2025) has supported this reciprocal interaction, showing that as students' digital game addiction increases, their psychological resilience levels decrease.

Analyses have revealed that psychological resilience can be associated with the risk of digital addiction and mental health problems in university students. Indeed, psychological resilience is directly and positively correlated with indicators of mental health (Hu et al., 2015; Ungar and Theron, 2020; van der Meulen et al., 2018). Empirical research confirms a strong relationship between psychological resilience and mental health (Färber and Rosendahl, 2018), underpinning the protective role of psychological resilience for mental wellbeing (Afek et al., 2021; Güldaş and Karsli, 2023).

Mediation analyses revealed that both digital addiction and psychological resilience partially mediate the emotional intelligence-mental health relationship. Therefore, emotional intelligence can be associated with university students' mental health both directly and indirectly through digital addiction and psychological resilience. In other words, higher levels of emotional intelligence can be directly linked to reduced digital addiction and lower risk of mental health problems. In addition, university students' psychological resilience can influence the strength of this relationship. Emotional intelligence can directly support mental health by strengthening individuals' abilities to recognize, regulate, and cope with stress (Ciarrochi et al., 2002; Shabani et al., 2010; Faghirpour et al., 2011; Zeidner et al., 2012). Psychological resilience can also partially mediate the relationship between emotional intelligence and psychological wellbeing, through its protective function in mental health (Akbari and Khormaiee, 2015; Jayalakshmi and Magdalin, 2015). Additionally, the more emotional intelligence strengthens psychological resilience, the better individuals respond to stressors (Armstrong et al., 2011; Li et al., 2015; Akbari and Khormaiee, 2015), which might result in reduced risk of mental health problems. On the other hand, individuals with high emotional intelligence are less likely to turn to digital environments as a coping mechanism (Yu and Zhou, 2021), which might result in higher risk of digital addiction (Zewude et al., 2024; Ahmed et al., 2025; Biolcati et al., 2025) with possible negative consequences on mental health.

In addition to the proposed research model, four alternative models were developed and tested to examine the relationships among variables and potential mediating effects. The analyses showed that the best model-data fit was achieved with the proposed model. In contrast, with the alternative Model 1, where digital addiction was the independent variable, the fit was only partial. These results indicate that although emotional intelligence is relatively more stable and fundamental than digital addiction, digital addiction may also be associated with university students' emotional intelligence levels. Although findings showing that individuals with internet addiction have relatively low levels of emotional intelligence (Juneja and Sethi, 2015) exist in the literature, this deserves and warrants further investigation to gain better insights.

## 6 Conclusion and implications

This study tested a theoretical model of the relationship between emotional intelligence and mental health problems of university students with a particular focus on how this relationship can be influenced by their digital addiction and psychological resilience. Findings have not only extended prior research that has typically investigated these variables in isolation but also offered a more comprehensive account of mental health functioning among university students by testing an integrated model of emotional, psychological, and behavioral factors within a single exploratory framework.

The results indicated that university students with higher levels of emotional intelligence are likely to experience fewer mental health problems since they are better at regulating their emotions, coping with the hassles of daily life, and recovering from stress. In addition, emotional intelligence not only functions as a personal resource that strengthens their adaptive coping capacities but also mitigates their maladaptive use of digital technologies through enhancing their self-regulation capacity. Similarly, the findings showed that university students' emotional intelligence can also enhance their mental wellbeing through fostering their psychological resilience. Taken together, these results highlight the importance of targeting both protective (i.e., emotional intelligence and psychological resilience) and risk factors (i.e., digital addiction) to enhance the mental wellbeing of university students.

The findings offer important implications for promoting university students' mental health. First, designing programs to enhance their emotion recognition, regulation, and empathy could be a promising entry point for preventive efforts. Second, interventions targeting psychological resilience through stress management, adaptive coping, and mindfulness-based programs may be particularly effective when integrated with emotional intelligence development. These interventions should also target promoting self-regulated digital engagement and help students develop healthier alternatives to emotionally driven, problematic use of digital technologies. Universities may consider embedding such programs within orientation courses, counseling services, or co-curricular activities.

## 7 Limitations

Despite the strengths of meta-analytical approaches in synthesizing empirical evidence, several limitations of the present study should be acknowledged. First, because the findings of the current study depend on data from published studies, any weaknesses in the design, measurement, or analysis of the primary studies may have influenced the pooled effect estimates. Second, due to variations in sample characteristics, research contexts, study designs, and measurement instruments, substantial heterogeneity is inherently observed in the dataset. Although we used a random-effects model to accommodate this variability, the sources of heterogeneity cannot be fully explained. Therefore, the results might not apply uniformly across contexts or populations. Furthermore, moderator analyses based on variables such as region, measurement instrument, or research design were not conducted to examine the sources of the high heterogeneity (I^2^ > 85%) observed in the pairwise relationships between variables because the main objective of the study was not to determine the sources of inter-study differences, but to test the theoretically proposed structural model and four alternative models. However, the adoption of the random effects TSSEM approach in the first stage allowed for the statistical reflection of inter-study variability in the pooled correlation matrix and the estimates in the second stage. It is recommended that future research contribute to the generalizability of the findings by systematically examining cultural, measurement, and design-based moderators. In addition, the findings reflect the state of the literature at the time of data collection and may not incorporate the most recent evidence. Similarly, the dataset might have missed some relevant research, although we employed a meticulous data search and extraction procedure.

## Figures and Tables

**Figure A1:**
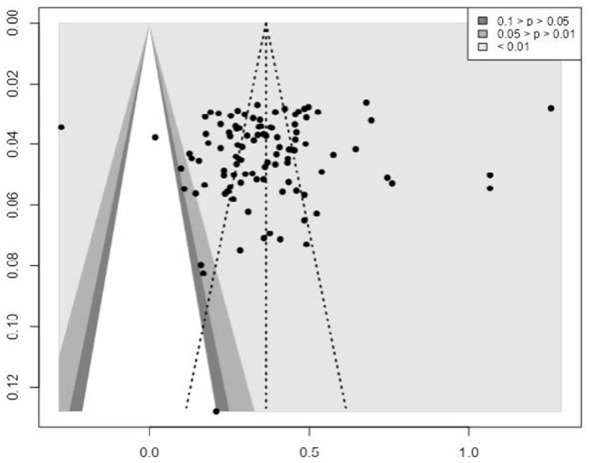
The funnel plot of the relationship between DA&MH.

**Figure A2:**
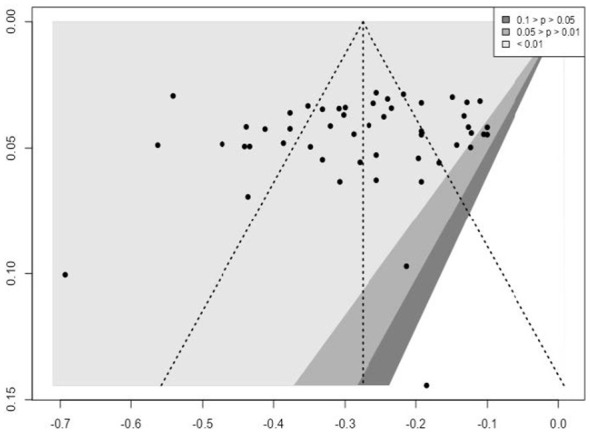
The funnel plot of the relationship between DA&PR.

**Figure A3:**
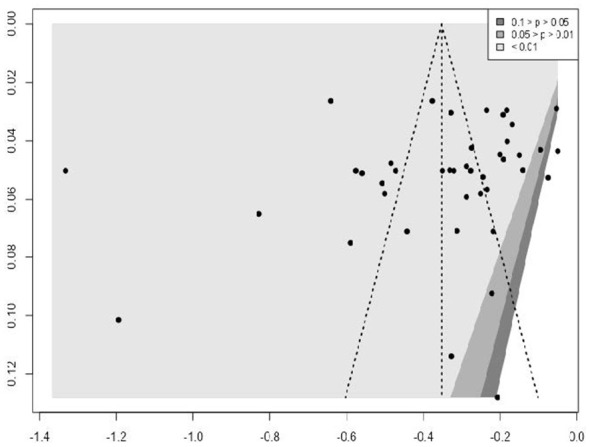
The funnel plot of the relationship between DA&EI.

**Figure A4:**
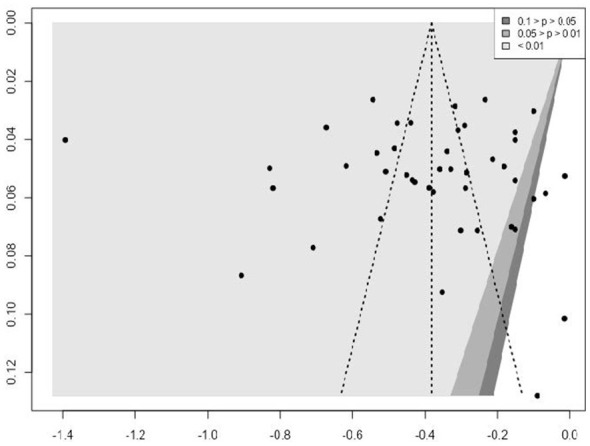
The funnel plot of the relationship between MH&EI.

**Figure A5:**
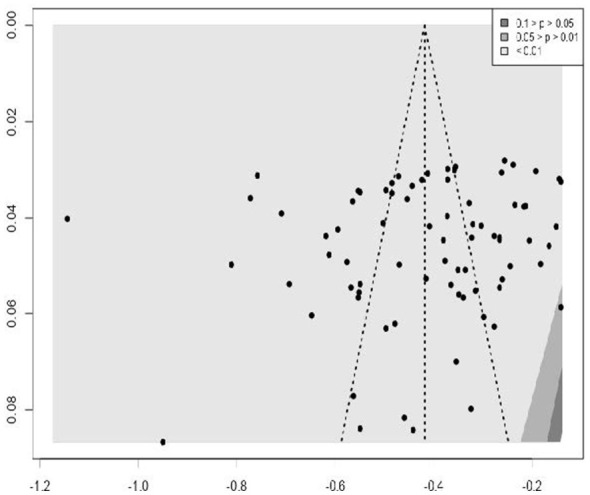
The funnel plot of the relationship between PR&MH.

**Figure A6:**
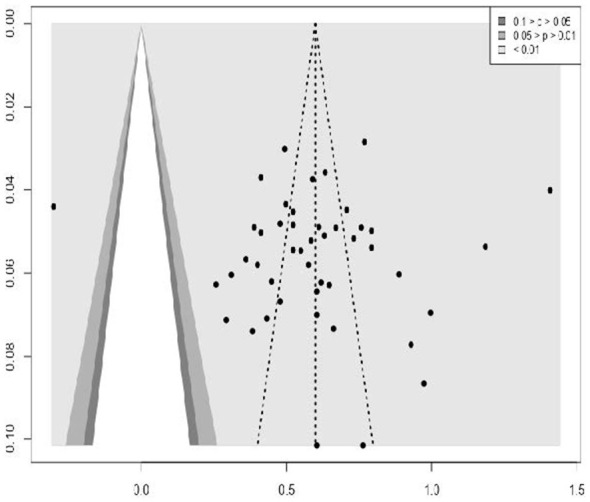
The funnel plot of the relationship between PR&EI.

## Data Availability

The original contributions presented in the study are included in the article/supplementary material, further inquiries can be directed to the corresponding author/s.
